# NELL-1, an Osteoinductive Factor, Is a Direct Transcriptional Target of Osterix

**DOI:** 10.1371/journal.pone.0024638

**Published:** 2011-09-13

**Authors:** Feng Chen, Xinli Zhang, Shan Sun, Janette N. Zara, Xuan Zou, Robert Chiu, Cymbelin T. Culiat, Kang Ting, Chia Soo

**Affiliations:** 1 Dental and Craniofacial Research Institute, University of California Los Angeles, Los Angeles, United States of America; 2 Department of Bioengineering, University of California Los Angeles, Los Angeles, United States of America; 3 School of Dentistry, University of California Los Angeles, Los Angeles, United States of America; 4 Oak Ridge National Laboratory, Oak Ridge, Tennessee, United States of America; 5 Orthopaedic Hospital, Department of Orthopaedic Surgery and the Orthopaedic Hospital Research Center, University of California Los Angeles, Los Angeles, United States of America; 6 School and Hospital of Stomatology, Peking University, Beijing, China; Institut de Génomique Fonctionnelle de Lyon, France

## Abstract

NELL-1 is a novel secreted protein associated with premature fusion of cranial sutures in craniosynostosis that has been found to promote osteoblast cell differentiation and mineralization. Our previous study showed that Runx2, the key transcription factor in osteoblast differentiation, transactivates the *NELL-1* promoter. In this study, we evaluated the regulatory involvement and mechanisms of Osterix, an essential transcription factor of osteoblasts, in *NELL-1* gene expression and function. Promoter analysis showed a cluster of potential Sp1 sites (Sp1/Osterix binding sites) within approximately 70 bp (from −71 to −142) of the 5′ flanking region of the human *NELL-1* transcriptional start site. Luciferase activity in our *NELL-1* promoter reporter systems was significantly decreased in Saos-2 cells when *Osterix* was overexpressed. Mutagenesis study demonstrated that this suppression is mediated by the Sp1 sites. The binding specificity of Osterix to these Sp1 sites was confirmed in Saos-2 cells and primary human osteoblasts by EMSA *in vitro* and ChIP assay *in vivo*. ChIP assay also showed that Osterix downregulated *NELL-1* by affecting binding of RNA polymerase II to the *NELL-1* promoter, but not by competing with Runx2 binding to the OSE2 sites. Moreover, *NELL-1* mRNA levels were significantly decreased when *Osterix* was overexpressed in Saos-2, U2OS, Hela and Glioma cells. Correspondingly, knockdown of *Osterix* increased *NELL-1* transcription and osteoblastic differentiation in both Saos-2 cells and primary human osteoblasts. These results suggest that Osterix is a direct transcriptional regulator with repressive effect on *NELL-1* gene expression, contributing to a delicate balance of regulatory effects on *NELL-1* transcription with Runx2, and may play a crucial role in osteoblast differentiation and mineralization. These findings also extend our understanding of the molecular mechanism of Runx2, Osterix, and NELL-1 and demonstrate their crosstalk during osteogenesis.

## Introduction

Through promoter analyses, we recently established NELL-1, a Nel-like molecule-1 [1], [2], as a novel direct transcriptional target of runt homology domain transcription factor-2 (Runx2) [3]. Site-directed mutagenesis and chromatin immunoprecipitation (ChIP) assays revealed at least three functional consensus osteoblast specific binding elements 2 (OSE2) on the human *NELL-1* promoter. Significantly, the overexpression of *NELL-1* was originally found in pathologically fusing and fused sutures in nonsyndromic unilateral coronal synostosis (UCS) patients [4], and *CMV*-*Nell-1* overexpression mice exhibited CS-like phenotypes that ranged from simple to compound synostoses [5]. These findings highly suggest that NELL-1 is a CS-associated factor with preferential osteogenic effects on cells of the osteochondral lineage. Furthermore, N-ethyl-N-nitrosourea (ENU)-induced *Nell-1* deficient mice revealed major abnormalities in the skeletal system such as decreased calvarial bone mineralization and decreased vertebral disc volume, and perinatal death due to respiratory failure secondary to a deformed cartilaginous ribcage [6]. This *Nell-1* deficient mouse model in addition to the overexpression transgenic mouse model further supports the critical role of Nell-1 in the Runx2 regulatory network of osteogenesis, however, the precise mechanism of action of Nell-1 remains unknown [7], [8].

Osterix/Sp7 (Osx), a member of the Sp1 transcription factor family, is also essential for osteoblastogenesis [9], [10], [11]. Like Runx2-null mice, Osterix-null mice exhibit complete absence of bone matrix and osteoblasts, indicating an absolute requirement for Osterix in osteoblast formation [9]. However, Osterix-null mice exhibit normal cartilage hypertrophy while Runx2-null mice do not. In addition, Osterix-null mice exhibit normal Runx2 levels, while Osterix is not expressed in Runx2 null-mice suggesting that Osterix is downstream of and tightly regulated by Runx2. The *Osterix* promoter does contain at least one functional Runx2 binding site [12], however, *Osterix* can be induced by BMP2 in Runx2-null cells [13], possibly through upregulation of Dlx5 and its phosphorylation by p38. Thus, Osterix exhibits both Runx2 dependent and independent regulation. Previous studies have suggested that Osterix functionally segregates osteoblast and chondrocyte lineages whereby bipotential precursor cells initially express Runx2 and then express Osterix to suppress chondrogenic lineage and promote osteoblast differentiation [14]. Consistent with this, Kaback et al. demonstrated Osterix expression in perichondrium, immature chondrocytes, and osteoblasts, but not hypertrophic chondrocytes during development [15].

Interestingly, the transduction of *AdNell-1* inhibited *Osterix* mRNA expression without affecting *Runx2* mRNA levels during osteoblastic differentiation of preosteoblastic MC3T3 cells [5], [16], which may indicate a potential regulatory and functional relationship between Nell-1 and Osterix in addition to what has been discovered between Nell-1 and Runx2 in osteoblastic differentiation, leading us to pursue this current study. Here we demonstrated that overexpression of Osterix can suppress *NELL-1* expression at the transcriptional level in multiple human osteoblast-like and non-osteoblastic cell lines, and verified that this inhibitory effect on NELL-1 expression with and without *Runx2* induction involves Osterix direct binding of Sp1 sites in the *NELL-1* promoter in a human osteosarcoma cell line, Saos2. We also verified that Nell-1 has inhibitory effects on *Osterix* expression during osteoblastic differentiation reciprocally. Taken together, we conclude that a delicate balance of regulatory effects on *Nell-1* transcription by Osterix and Runx2 is crucial, and these novel findings provide new insights into the underlying mechanism of Nell-1′s action during osteochondral differentiation.

## Materials and Methods

### Cell Culture

Saos-2 cell line was purchased from ATCC (Manassas, VA) and primary human osteoblast cells (HOb) were purchased from Cell Applications Inc (San Diego, CA). Cells were cultured in growth medium of Dulbecco's modified Eagles' medium (DMEM) with 10% fetal bovine serum (FBS), 100 units/ml penicillin, and 100 µg/ml streptomycin or osteoblastic differentiation medium containing additional 50 ug/ml ascorbic acid and 10 mM beta-glycerophosphate in the growth medium. Cells were plated at a density of 2.5×10^4^ cells/cm^2^ in DMEM for the experiments.

### Reporter and Expression Constructs

The p2213WT-Luc and p325WT-Luc vectors containing the hum*an NELL*-1 promoter fragment were generated as described previously [3]. Mutant constructs of the p325WT-Luc promoter including p325mut all-Luc with mutation in all Sp1 sites of both cluster Site A and Site B, p325mutSiteA-Luc with mutation in cluster Site A, and p325mutSiteB with mutation in Site B were produced by *GenScript USA Inc.* (Piscataway, NJ). Human *Osterix* expression construct (pcDNA3.1-Osterix, pOsx), generated by RT-PCR from Saos-2 cell cDNA, and empty pcDNA3.1 control vector (pCtr) were utilized for all co-transfection experiments. The Runx2 expression plasmid (pRunx2) was a generous gift from Dr. Wenfang Wang of Harvard University. Renilla control plasmid was purchased from Promega (Madison, WI). All constructs were confirmed by reproducible sequencing (Laragen, Los Angeles, CA). Each experiment was repeated at least 3 times. The data is presented as the mean ± SD. A t-test was performed, with p<0.05 considered statistically significant [3].

### Transient Transfection and Reporter Assay

Cells in 6 well plates were transfected and grown to 80–90% confluence with Lipofectamin 2000 (Invitrogen, Carlsbad, CA). For each reaction, the plasmid DNA (promoter-luciferase vectors p2213WT-Luc or p325WT-Luc plus Renilla plasmid, with pOsx or pCtr constructs) was transfected according to the manufacturer's instructions. Forty-eight hours after transfection, the cultures were harvested, and luciferase activity was assayed using Dual-Luciferase Assay System (Promega, Madison, WI), and then normalized by co-transfected Renilla activity. Each experiment was repeated at least 3 times. The data is presented as the mean ± SD. A t-test was performed, and p<0.05 was considered statistically significant [3]. Total RNA of transfected cells was isolated by Trizol Reagent (Invitrogen, Carlsbad, CA), and the expression levels of *NELL-1* and other genes were detected by real-time PCR.

### Electrophoretic Mobility Shift Assays

**Figure 1:**
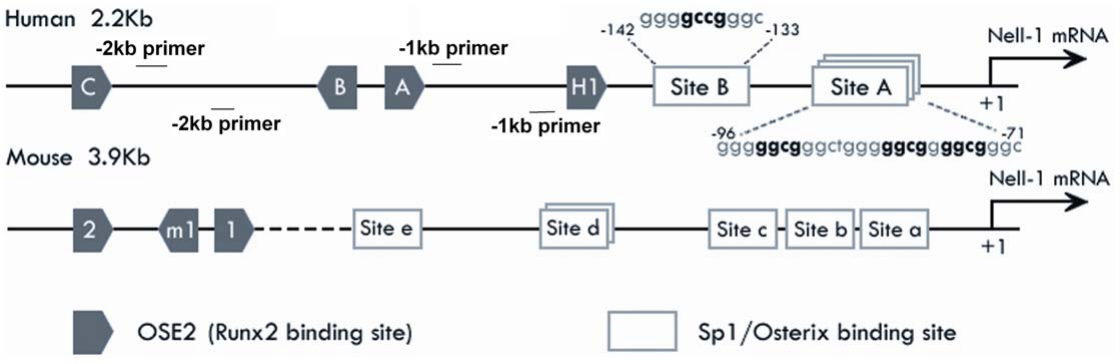
Schematic of the human and mouse *Nell-1* promoters (not drawn to scale). *In silico* analysis identified a cluster of potential Sp1/Osterix binding sites within approximately 70 bp of the 5′ flanking region of the human *NELL-1* gene. Within the cluster, Site A contains three overlapping potential Sp1 sites from −71 to −96 bp and Site B contains a single Sp1 site from −133 to −142 bp. Four OSE2 sites to which Runx2 binds in human *NELL-1* promoter are also shown (labeled A, B, C and H1). A similar pattern is seen in mouse *Nell-1* promoter. Multiple putative Sp1 sites (labeled Site a∼e) are located before three OSE2 sites (labeled 1, m1 and 2). The relative location of primers for CHIP assay are indicated on the human 2.2 kb promoter.

Preparation of nuclear extracts from Saos-2 cells transfected with pCtr or pOsx were performed using Nuclear Extract Kit (Pierce, Rockford, IL) according to the manufacturer's protocol. Briefly, cells were harvested in ice-cold PBS supplemented with phosphatase and protease inhibitors, then lysed in hypotonic buffer and 1% Triton X-100. After collection of the cytoplasmic fraction, nuclei were lysed and the nuclear extract proteins were solubilized in lysis buffer supplemented with 10 mM DTT, and protease inhibitor cocktail. According to the human *NELL-1* promoter analysis (**Fig. 1**), we designed two oligos, Site A containing three overlapping Sp1 sites and Site B containing a single Sp1 site. The mutated Site A oligo (Mut^A^) has a mutation in every Sp1 site while the mutated Site B oligo (Mut^B^) has the same mutation at its Sp1 site. Oligonucleotide probes labeled with and without biotin were generated from Invitrogen. Sequences used include: Site A: 5′-GCGGTGGGGGGCGGGCTGGGGGCGGGGCGGCCGGGGCTG-3′, Mut^A^: 5′-GCGGTGGGGGAAGGGCTGGGGAAGGGAAGGCCGGGGCTG-3′, Site B: 5′-GCCGCAGCGGGGGGCCGGGCCCCGAGCCCC-3′, and Mut^B^: 5′-GCCGCAGCGGGGGGAAGGGCCCCGAGCCCC-3′. The EMSA protocol followed the LightShift Chemiluminescent EMSA Kit (Pierce, Rockford, IL) instructions. Briefly, 2 µg of nuclear extract protein (NE) plus biotin labeled oligonucleotide with and without anti-Osterix (Abcam, Cambridge, MA) in 20 µl binding system (containing 2.5% glycerol, 5 mM MgCl_2_, 50 ng/µl poly (dI·dC), 0.05% NP-40 and 1x binding buffer) was incubated at room temperature for 20 minutes. Five µl of 5x loading buffer was added to each 20 µl binding reaction, then 20 µl of each sample was loaded onto the 5% polyacrylamide gel in 0.5x TBE. Samples were electrophoresed until the bromophenol blue dye had migrated approximately 3/4 down the length of the gel. The gel and nylon membrane were sandwiched in a clean electrophoretic transfer unit, and transferred at 380 mA (∼100V) for 30 minutes. Then DNA and membrane were crosslinked using a UV-light cross-linker instrument. Biotin-labeled DNA was detected by Chemiluminescence provided by the LightShift Chemiluminescent EMSA Kit (Pierce, Rockford, IL). The unlabeled oligos of Site A and Site B at 20x and 200x or 200x mutated Site A and Site B were added to compete specifically with labeled oligo binding in the competitive EMSA.

### Chromatin Immunoprecipitation Assays

Saos-2 cells and primary human osteoblasts were used for chromatin immunoprecipitation (ChIP) assays with the One-day ChIP-qPCR kit (SABiosciences, Frederick, MD) according to the manufacturer's instructions. Briefly, cells were fixed with 1% formaldehyde for 10 minutes at 37°C. Cross-linked lysates were treated with glycine to stop the fixation reaction. Lysates were resuspended in shearing buffer and then sonicated to shear chromatin. Cross-linked lysates were immunoprecipitated with anti-Osterix (Abcam, Cambridge, MA), anti-Runx2 (Santa Cruz Biotechnology, Inc, Santa Cruz, CA), or non-specific IgG (SABiosciences, Frederick, MD) antibodies. Then DNA was recovered and used for qPCR with *NELL-1* promoter primer pairs designed and provided by SABiosciences following the manufacturer's instructions. The human RNA Polymerase II ChIP-grade antibody and human GAPDH ChIP-qPCR control primer were provided in the ChampionChIP kit. The *NELL-1* -1 kb primer set (NM_006157.2 −01 kb) covers 1 kb proximal promoter region where all Sp1/Osterix binding sites and three OSE2 sites are located, and the *NELL-1* −2 kb primer set (NM_006157.2 −02 kb) covers the *NELL-1* promoter region from −1 kb to −2 kb where no Sp1 site and one OSE2 site exists (**Fig. 1**). Each experiment was repeated at least 3 times. The data is presented as the mean ± SD. A t-test was performed, with p<0.05 considered statistically significant [3].

### Reverse Transcription-PCR and Real-Time PCR

One µg of DNase I treated total RNA were used for reverse transcription. The ABI Prism 7300 Real Time PCR System was utilized to quantify gene expression in the exponential phase of PCR reactions. TaqMan primer-probe sets for *osteocalcin (Ocn)*, *osteopontin* (*Opn*), *NELL-1*, *Osterix*, and *glyceraldehyde-3-phosphate dehydrogenase* (*Gapdh*) were purchased (Applied Biosystems, Foster City, CA) and analyzed by real time PCR as previously described [5]. Relative gene expression profiles were calculated using the comparative quantification formula as 2^−ΔΔ^Ct based on the evaluation of similar dynamic ranges for RT-PCR efficiency of both *Gapdh* and the target genes. Each experiment was repeated at least 3 times. The data is presented as the mean ± SD. A t-test was performed, with p<0.05 considered statistically significant [3].

### Transfection of *Osterix* siRNA into Saos-2 cells and primary human osteoblasts

Human *Osterix* siRNA oligos were designed and synthesized by Invitrogen (Silencer Select pre-designed siRNA s42458, s42459 and s42460 against *Osterix* mRNA). Saos-2 cells or human osteoblasts were seeded at 2.5×10^4^/cm^2^ into 6 well plates and allowed to reach 50% confluence the following day for siRNA transfection. Thirty pg/well of control siRNA or Osterix siRNA oligos mixture per well was added with the Lipofectamin 2000 reagent (Invitrogen, Carlsbad, CA) following the manufacturer's instructions. The blocking efficacy for the expression of human *Osterix* mRNA was measured at 2 days and 7 days post-transfection with real time PCR. Each experiment was repeated at least 3 times. The data is presented as the mean ± SD. A t-test was performed, with p<0.05 considered statistically significant [3].

## Results

### *NELL-1* promoter contains multiple putative Sp1 sites (Sp1/Osterix binding sites)

*In silico* analysis of the human *NELL-1* promoter identified a cluster of potential Sp1 sites (Sp1/Osterix binding sites) within approximately 70 bp of the 5′ flanking region of the human *NELL-1* gene (from −71 to −142 bp upstream of the *NELL-1* transcriptional start site) (www.genomatix.de). Within this cluster, Site A contains three overlapping potential Sp1 sites from −71 to −96 and Site B contains a single Sp1 site from −133 to −142 (**Fig. 1**). In comparison to the human *NELL-1* promoter, the mouse *Nell-1* promoter also contains multiple putative Sp1 sites that reside at −107, −149, −195, −524, −536 and −971 bp upstream of the transcriptional start site. Similarly, a cluster of Sp1 sites was also identified by virtue of their proximity and overlapping consensus sequences between the fourth and fifth Sp1 sites (**Fig. 1**). To better illustrate the spatial distribution of Sp1 sites in both human and mouse *Nell-1* promoters, the locations of previously identified Runx2 binding sites OSE2 are also displayed in **Fig. 1**, with three functional sites labeled A, B, C and a cryptic site H1 in the human *NELL-1* promoter, and two sites labeled site 1 and 2 and a cryptic site m1 in the mouse promoter region. The presence of multiple Sp1 binding sites in the *NELL-1* promoter across species of mouse and human is a good indication that *NELL-1* transcription may also be directly regulated by Osterix in addition to Runx2.

### Human *NELL-1* promoter is responsive to *Osterix* by reporter assay

**Figure 2:**
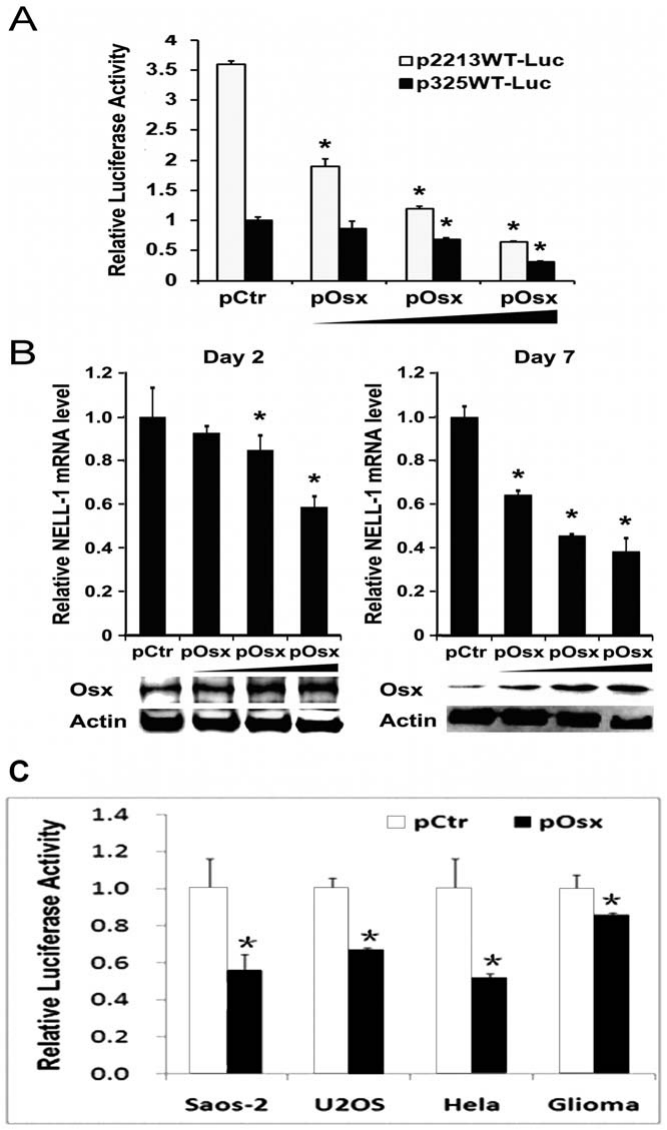
Dose-dependent *NELL-1* expression response to Osterix. (**A**) Human *NELL-1* promoter is responsive to Osterix in a dose-dependent manner shown by reporter assay. Graph detailing the luciferase activity in Saos-2 cells co-transfected with either the p2213WT-Luc or the p325WT-Luc promoter-luciferase constructs as well as 0, 0.25, 0.5, or 1 ug of Osterix expression vector (pOsx). Data are expressed as a percentage of the luciferase activity of the p325WT-Luc construct in the absence of control vector (pCtr). (**B**) Osterix overexpression decreased *NELL-1* mRNA level in Saos-2 cells at 2 days and 7 days post-transfection. Western blot showed Osterix protein levels at 2 days and 7 days post-transfection. (*p<0.05) (**C**) Osterix transcriptional repression of NELL-1 promoter in osteoblastic and non-osteoblastic cells. A graph detailing the luciferase activity of p325WT-Luc 2 days post-transfection in Saos-2, U2OS, Hela and Glioma cell lines co-transfected with pCtr or pOsx. (*p<0.05)

To investigate whether the Sp1 sites contribute to *NELL-1* transcription, we used the human *NELL-1* promoter reporter systems −2213bp (p2213WT-Luc) and −325bp (p325WT-Luc) as published [3]. These systems contain a 2213 bp or 325 bp length of genomic DNA upstream of the human *NELL-1* transcription start site. We confirmed the presence of these putative Sp1 sites at predicated positions by full-length sequencing (Laragen, LA, CA) (**Fig. 1**). Co-transfection experiments were performed to examine a dose–dependent response for both promoter constructs in Saos-2 cells. The Saos-2 cell line was chosen as a suitable system to study the human promoter and was previously used to study human *NELL-1* promoter OSE2 sites [3]. Saos-2 cells have endogenous expression of both *Osterix* [17] and *NELL-1*, and have the necessary osteoblast-related factors for differentiation and formation of mineralized bone nodules [18], [19]. Saos-2 cells transfected with the p2213WT-Luc and 0.25 µg to 1 µg *Osterix* expression vector (pOsx) had up to 80% decrease in luciferase activity in a dose-dependent manner compared to Saos-2 cells transfected with the p2213WT-Luc and control vector (pCtr) 2 days after transfection. Although the basal luciferase activity is reduced in the truncated p325WT-Luc construct group since it lacks 3 consensus OSE2 sites [3], the truncated construct with pOsx showed a similar percent decrease compared to control vector (**Fig. 2A**), indicating that the short fragment containing these Sp1 sites in the p325WT-Luc construct are responsible for Osterix to down-regulation of luciferase activity.

### Osterix forced expression decreases *NELL-1* mRNA levels in human osteoblast-like and non-osteoblastic cells

To investigate whether Osterix controls endogenous *NELL-1* gene expression, Saos-2 cells were transiently transfected with either an empty vector or different amounts of Osterix expression constructs for 2 days and 7 days. Saos-2 cells were chosen for this analysis because we have previously shown that the high expression level of *NELL-1* in these cells can induce several osteoblastic maker genes such as *Opn* and *Ocn* and promote their differentiation and mineralization [3]. The Osterix protein levels in Saos-2 cells transfected with different amounts of pOsx were detected by Western blot (Fig. 2B). In this study, the forced expression of *Osterix* in Saos-2 cells significantly decreased *NELL-1* expression at 2 days post-transfection in a dose–dependent manner, and continued to reduce the levels for up to 7 days (Fig. 2B).

In addition to the Saos2 cells, the transcriptional repression of Nell-1 promoter by Osterix was also detected in other human cell lines including an immature osteoblast, U2OS cells and two non-osteoblastic cell lines, Hela and Glioma cells (**Fig.** **2C**). Similar results in these cell lines compared to results in Saos2 cells further suggest that the transcriptional repression of *NELL-1* promoter by Osterix exists in human cells irrespective of tissue origin or degree of osteoblastic maturity.

### Sequence specific binding of Osterix to Sp1 sites within the *NELL-1* promoter

**Figure 3:**
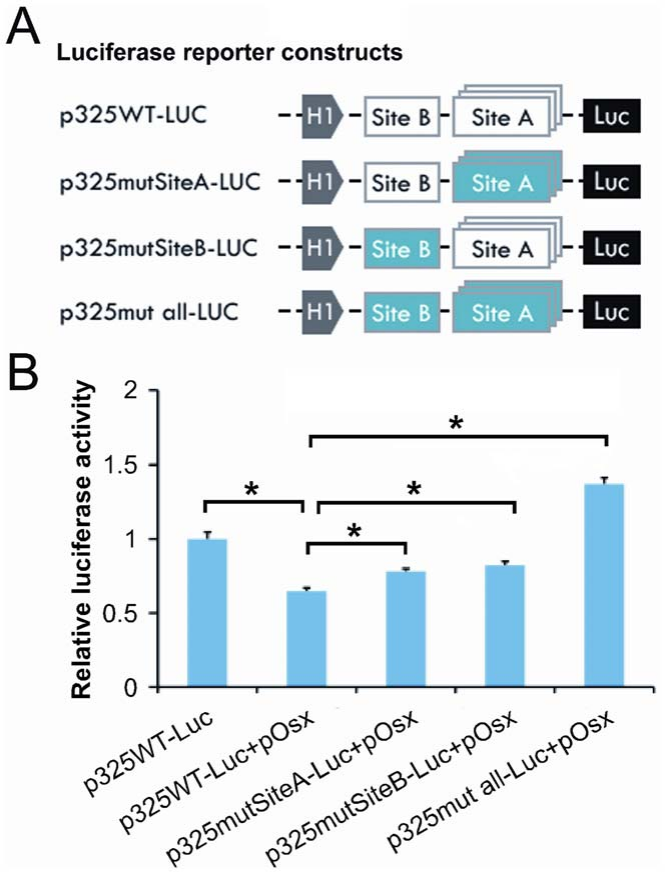
Both Site A and Site B are functional for Osterix-mediated *NELL-1* promoter activity suppression. (**A**) Schematic of p325WT-Luc, p325mut all-Luc, p325mutSiteA, and p325mutSiteB constructs. Blue represents mutated Sp1 sites. (**B**) Graph depicting promoter activity on Saos-2 cells cotransfected with pOsx as well as p325WT-Luc or mutated *NELL-1* promoter-luciferase constructs (p325mut all-Luc, p325mutSiteA-Luc, and p325mutSiteB-Luc, respectively). Data are reported as percent activity of control cells transfected with pCtr. (*p<0.05)

Since all these Sp1 sites lie within the 325bp promoter of the proximal *NELL-1* transcriptional start site, to determine the functional relevance of all Sp1 sites in Osterix-mediated decrease of *NELL-1* promoter activity, we generated a mutant promoter construct (p325mut all-Luc, Genscript Co, Piscataway, NJ) containing an alteration of the Sp1 sites by point mutation known to disrupt Osterix binding [9] (**Fig. 3A**). This mutant construct, p325mut all-Luc with mutations in all Sp1 sites of both cluster Site A and Site B, was transfected into Saos-2 cells and the downstream reporter gene luciferase activity was analyzed with and without forced *Osterix* expression. The Osterix-induced suppression of luciferase activity was statistically significant in the wild type construct p325WT-Luc (p<0.05) (Fig 3B). Furthermore, the complete suppression of Osx inhibitory effect was observed in the p325mut all-Luc construct as compared to p325WT-Luc in the setting of Osterix overexpression (p<0.05). This result strongly indicates that these Sp1 sites of the Nell-1 promoter are required for Osx binding in regulating *NELL-1*'s transcription.

To determine which Sp1 site is more important to induce the suppression, we made two additional mutant reporter constructs, p325mutSiteA-Luc with mutations in cluster Site A, and p325mutSiteB with mutation in Site B. Notably, the suppression of luciferase activity by expression of Osterix was still observed when either p325mutSiteA or p325mutSiteB constructs were used. However, the levels of Osterix overexpression-mediated suppression in these constructs were significantly diminished in comparison to p325WT construct (p<0.05**)** (**Fig. 3B**). These results indicate that both Site A and Site B have functional roles in the suppression of *NELL-1* when bound by Osterix, and both are absolutely necessary and responsible for the complete suppression of Osx inhibitory effect on *NELL-1's* transcription when they are mutated simultaneously (p<0.05).

To further examine DNA-protein interactions at these Sp1 sites, EMSAs using Saos-2 nuclear extracts and the respective oligonucleotide probes containing the Sp1 sites were performed. Since these Sp1 sites were within 70 bp in the *NELL-1* proximal promoter, we divided them into two respective oligonucleotide probes in our experiment—Site A (from −101 to −62 bp upstream of *NELL-1)* containing three overlapping potential Sp1 sites and Site B (from −153 to −124 bp upstream of *NELL-1* transcriptional start site) containing a single Sp1 site.

**Figure 4:**
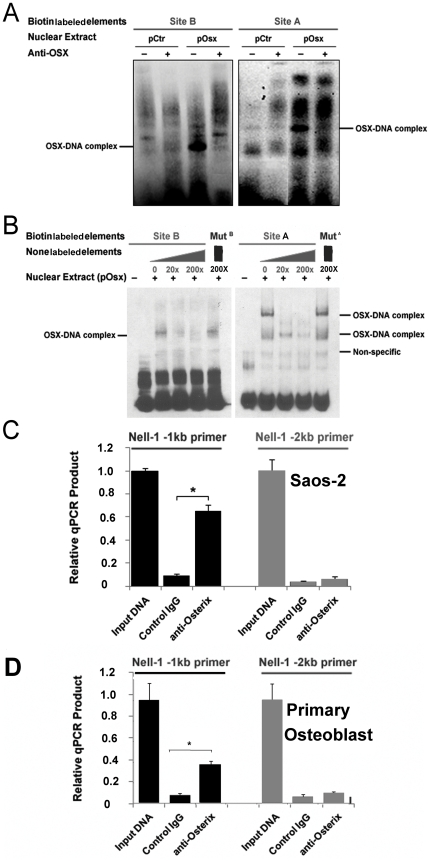
Osterix specifically binds to Sp1 sites in the *NELL-1* promoter *in vitro* and *in vivo.* (**A**) EMSA of Saos-2 nuclear proteins transfected with pCtr or pOsx binding to SiteA (containing three proximal Sp1 sites) and SiteB probes. Supershifts with specific Osterix antibody indicate the specific Osterix-DNA complexes. (**B**) EMSA depicting primary human osteoblast cell (hOB) nuclear proteins transfected with pOsx binding to the SiteA probes, with competition by 20x and 200x unlabeled SiteA and 200x unlabeled mutated SiteA (MutA) oligonucleotides. Note that the MutA probes failed to bind nuclear proteins. The same pattern is also seen by competition of SiteB element and MutB. (**C–D**) Osterix binds to endogenous Sp1 sites of the human *NELL-1* promoter in Saos-2 (C) and hOB (D) cells. The *NELL-1* -1 kb primer set covers 1 kb proximal promoter region containing all Sp1/Osterix binding sites and three OSE2 sites. The *NELL-1* -2 kb primer set covers *NELL-1* promoter region from −1 kb to −2 kb where no Sp1/Osterix binding site but one OSE2 site exists. The qPCR products depict DNA amplified from Chromatin Immunoprecipitation with cells utilizing Control IgG and Osterix antibody. Input DNA represents positive genomic DNA control.

Since Tomohiro reported that Sp1/Sp3 can also occupy the Sp1 sites in Saos-2 cells [17], we force expressed Osterix in Saos-2 cells to increase its amount in the nuclear extract (NE) for gel shift assay. We were then able to find the main protein-DNA (Osterix-Site A and Osterix-Site B) complex bands in the two groups with and without Osterix overexpression (**Fig. 4A**). These protein-DNA complex bands were confirmed by super shift assay when Osterix antibody was added (**Fig. 4A**).

Furthermore, specificity of binding to the Sp1 site was confirmed by competition analysis with WT probes and by mutant probes containing 2 bp mutations known to disrupt Osterix binding described in mutant reporter systems. Incubation of NE from Saos-2 cells transfected with pOsx led to the formation of protein–DNA complexes with WT oligonucleotides. In contrast, the oligonucleotides harboring the 2 bp mutations had no protein-DNA complexes detected (**Fig. 4B**). Binding of the promoter sites by labeled WT probe was diminished with the addition of 20x and 200x unlabeled WT oligonucleotide, but no effect was seen with the addition of 200x unlabeled mutated oligonucleotides (**Fig. 4B**). These results demonstrate that Osterix is indeed part of the DNA-protein complex in the *NELL-1* promoter and indicate that NE from Saos-2 cells has Osterix that can specifically bind to the Sp1 sites of the human *NELL-1* promoter.

### Osterix binding to the endogenous *NELL-1* promoter *in vivo*

Since our reporter assay and EMSA studies described above indicate that Osterix protein recognized these Sp1 sites in the human *NELL-1* promoter, we next examined whether Osterix is able to interact directly with the *NELL-1* promoter *in vivo*. To study this we used ChIP-qPCR assay (ChampionChIP One-Day kit, SABiosciences, Frederick, MD) to examine the binding of Osterix to the *NELL-1* promoter region in Saos-2 cells. The qPCR products between anti-Osterix and control IgG showed a significant difference in the *NELL-1* promoter −1 kb group, but the difference was not seen in the *NELL-1* promoter −2 kb group (**Fig. 4C**), suggesting that Osterix can directly interact with *NELL-1* promoter *in vivo* and specifically bind to the chromatin fragment containing these Sp1 sites region covered by specific *NELL-1* promoter −1 kb qPCR primer. To further confirm this finding we also performed the ChIP-qPCR assay in primary human osteoblast cells. The results indicated the existence of similar recessive regulatory relationship of Osterix and Nell-1 through direct binding of Osterix to Sp1 sites of Nell-1 promoter (**Fig. 4D**). These results further verify that endogenous Osterix both in Saos-2 cells and primary human osteoblast specifically associate with these Sp1 sites in the endogenous human *NELL-1* promoter, and that Osterix is directly involved in the regulation of *NELL-1* gene activity through its physical interaction with the *NELL-1* promoter.

### Osterix down regulates *NELL-1* expression without disruption of Runx2 binding to *NELL-1* promoter OSE2 sites

**Figure 5:**
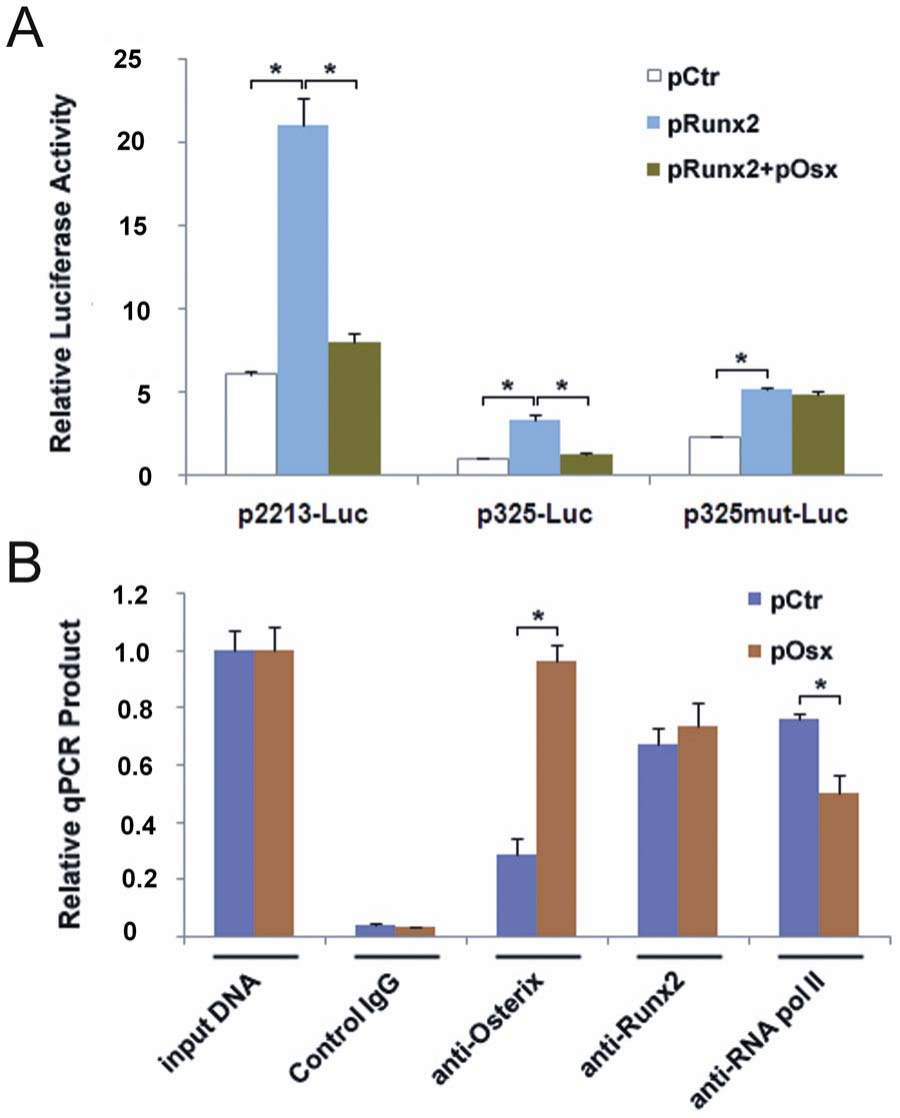
Osterix down regulates Runx2-induced *NELL-1* promoter activity. (**A**) Graph depicting promoter activity in Saos-2 cells after co-transfection with control empty pcDNA3.1 vector (pCtr), pcDNA-Runx2 (pRunx2) or pRunx2 plus pOsx expression vectors as well as p2213-Luc, p325-Luc or p325mut-Luc *NELL-1* promoter-luciferase constructs. Data are reported as fold changes in comparison to control cells transfected with pCtr and p325WT-Luc constructs. (p<0.05) (**B**) Osterix affects binding of RNA polymerase II to *NELL-1*'s promoter, but does not compete with Runx2 binding of OSE2 sites. The *NELL-1* -1 kb primer set used covers 1 kb proximal promoter region where all Sp1/Osterix binding sites and three OSE2 sites are located. The qPCR products depict DNA amplified from Chromatin Immunoprecipitation with Saos-2 cells transfected with pCtr or pOsx utilizing Control IgG, Osterix antibody, Runx2 antibody or RNA polymerase II antibody. Input DNA represents positive genomic DNA control. (*p<0.05)

Because our previous study showed that Runx2 positively regulates *NELL-1* expression through three consensus and one cryptic OSE2 site (H1) in the *NELL-1* promoter, here we co-transfected Osterix and Runx2 with p2213-Luc or p325-Luc constructs to detect Osterix effects on Runx2 induced *NELL-1* promoter activity. The reporter systems were co-transfected with pRunx2 and reporter constructs plus pOsx or pCtr. The luciferase activity after treatment with pOsx was significantly reduced compared to treatment with pCtr in both p2213WT-Luc and p325WT-Luc groups (**Fig. 5A**). This result suggests that high level Osterix can suppress Runx2 induced *NELL-1* expression. In order to determine whether this suppression is mediated through these Sp1 sites, we used the construct (p325mut all-Luc) with all mutated Sp1 sites disrupting Osterix binding (**Fig. 3A**). As expected, in the p325mut all-Luc group, there was no difference in luciferase activity between pOsx and pCtr (**Fig. 5A**) indicating that the suppression of Runx2 induced *NELL-1* expression by Osterix requires functional Sp1 sites.

Our previous *NELL-1* promoter analysis also showed that these Sp1 sites (−71∼−142 bp) are located proximal to the Runx2 OSE2 binding site (H1, −247 bp). It is possible that Osterix down regulation of *NELL-1* promoter activity is mediated by suppression of Runx2 binding to the H1 site. Therefore, ChIP-qPCR assay was used to detect binding between Runx2 and *NELL-1* promoter with and without Osterix forced expression. The same amount of chromatin was used for ChIP assay plus control IgG, Osterix antibody, Runx2 antibody and general transcriptional factor RNA polymerase II antibody. ChIP-qPCR products were normalized by endogenous GAPDH amounts between Osterix transfection and control vector groups. The results showed that Osterix binding to *NELL-1* promoter was significantly increased in the Osterix forced expression group compared to control vector group. There was no obvious difference seen in Runx2 binding to *NELL-1* promoter with and without Osterix forced expression (**Fig. 5B**). Interestingly, the general transcription factor RNA polymerase II binding to *NELL-1* promoter was significantly decreased in the Osterix overexpression group (**Fig. 5B**), indicating one possible mechanism for Osterix negative regulation of *NELL-1* promoter activity.

### Osterix siRNA increases *NELL-1* mRNA levels in Saos-2 cells concomitantly with increased mineralization

**Figure 6:**
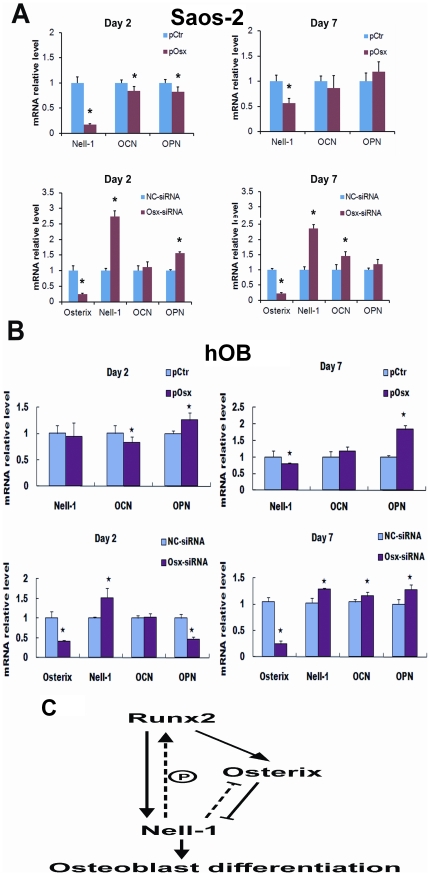
Osterix affects *NELL-1* and some bone marker genes expression in Saos-2 cells (A) and primary human osteoblast (hOB) (B). Real time PCR analysis of *Nell-1*, *OCN* and *OPN* transcripts in Saos-2 cells transiently transfected with Control (pCtr) or Osterix expression vector (pOsx) at 2 days and 7 days (top panel). The effects with transcient transfection of Negative control siRNA (NC-siRNA) or *Osterix* siRNA mixture (Osx-siRNA) were revealed by Real time PCR analysis at 2 days and 7 days (bottom panel). (**C**) Diagram of the regulatory relationship between Runx2, Osterix and NELL-1. Runx2 positively regulates Osterix and NELL-1. Osterix negatively controlled *NELL-1* expression in this study. NELL-1 was also shown to positively affect Runx2 through phosphorylation and negatively feedback on Osterix during osteogenesis.

The data showed that Osterix forced expression decreases *NELL-1* mRNA levels in Saos-2 cells (**Fig. 2B**). To further demonstrate the effect of suppression, we also analyzed other osteoblastic marker mRNA levels after Osterix overexpression in Saos-2 cells and primary human osteoblasts. Interestingly, some markers such as *Ocn* and *Opn* expression levels also decreased following the decrease of *NELL-1* expression at 2 days post-transfection (**Fig. 6A**). However, by 7 days post-transfection, *Ocn* and *Opn* expression levels showed no significant difference between the pCtr and pOsx groups in Saos-2 cells. Moreover, *Ocn* expression level also decreased in a similar fashion as Nell-1 at 2 days post-transfection in primary human osteoblasts (Fig. 6B), but *Opn* expression patterns were different between Saos-2 osteosarcoma cells and normal primary human osteoblast cells, which may indicate that overexpression of *Osterix* plays a transient and more complicated role with variable effects on bone marker gene levels at different stages of maturation of human osteoblasts.

To further confirm Osterix suppression of *NELL-1* expression, we inhibited *Osterix* mRNA level using siRNA in Saos-2 cells and primary human osteoblasts. Data showed that *NELL-1* mRNA levels increased almost 3 fold 2 days after *Osterix* siRNA transfection at which time *Osterix* mRNA expression levels were decreased by 80% in Saos-2 cells (**Fig. 6A**). *Ocn* and *Opn* expression also increased slightly 2 days after transfection. At post-transfection day 7, when *Osterix* mRNA levels were still less than 30%, *NELL-1* mRNA levels continued to be elevated. *NELL-1* and *Ocn* mRNA levels also increased in a similar pattern at 7 days post-transfection (**Fig. 6A**). To further confirm Osterix regulation of *NELL-1* in mature osteoblast cells, these experiments were performed in human primary osteoblasts. Although the inhibition efficiency of Osx-siRNAs (around 50%) in this cell line is less than that in Saos-2 cells at Day 2, *NELL-1* mRNA levels showed significant increase along with significant changes in other bone markers (*Ocn* and *Opn*) after 7 days post *Osterix* siRNA transfection (Fig. 6B). Alizarin Red staining was also used to detect mineralization during osteoblast differentiation. *Osterix* siRNA transfection increased the mineralization of Saos-2 cells at 9 days post-transfection (data not shown), consistent with bone marker gene mRNA level increase in *Osterix* siRNA assay.

## Discussion

NELL-1 is a novel osteoinductive factor under direct transcriptional regulation of Runx2 [3], [5], the master transcription factor of osteogenesis [20]. Osterix is another essential transcription factor for osteoblast differentiation and bone formation directly downstream of Runx2 [12]. In this study we sought to determine the regulatory and functional relationship between these two downstream targets of Runx2, in particular to validate the functional characteristics of potential Osterix binding sites in the human *NELL-1* promoter revealed by in silico analysis. Our data showed that Osterix exhibits repressive instead of assumed inductive effect on *NELL-1* expression at the transcriptional level by binding directly to Sp1 sites in the *NELL-1* promoter region; a surprising finding given the fact that NELL-1 and Osterix are both considered pro-osteogenic factors [5], [9], [10], [16], [21]. This adds NELL-1 as a member of Osterix regulated molecules that include Col 1a [9], Col 11a2 [17], DKK1 [22] and IL-1a [23]. Like IL1-a, NELL-1 is also negatively regulated by Osterix. In addition, we also found that the Sp1 binding elements in the human *NELL-1* promoter, identified as two clusters, Site A and B, have similar capacity to be fully occupied by Osterix to mediate repression. The release of this repression can occur only when Site A and B are mutated simultaneously.

The definitive mechanisms underlying the activating or inhibitory effects of Osterix on target promoters of these molecules remain unclear. Interestingly, basic transcription element B1 (BTEB1), a Sp1-like protein, has been found to activate transcription on promoters containing multiple GC boxes but act as a repressor on promoters containing only a single GC box [24]. This differential effect on multiple versus single GC box in gene promoters also applies to Osterix direct targets including activation of Col 11a2 and DKK1 which both have multiple binding sites, and repression of IL-1a which has a single binding site. However, this rule does not apply to all targets of Osterix, as Col 1a which has a single binding site is activated, not repressed, by Osterix, while Nell-1 with multiple sites is repressed. Col 1a regulation is more complex, as its regulation has been reported to also involve NFATc1 as a co-factor that forms a complex with Osterix to bind the consensus Sp1 binding site [10]. It is possible that NFATc1 may modulate Osterix-mediated transactivation by recruitment of other transcriptional co-activators [10]. Most recently, another co-factor of Osterix, NO66, a Jumonji family histone demethylase, has been reported to impair transcriptional activation of Osterix through interaction with the Osterix activation domain. In particular, the interaction between Osterix and NO66 is believed to regulate Osterix target genes in osteoblasts through modulating histone methylation [25]. Osterix transcriptional repression of Nell-1, a gene expressed preferentially in osteoblasts, may therefore also involve a co-factor leading to the negative effect on *NELL-1* promoter activity.

Runx2 is known as the master regulator of osteochondrogenesis, promoting commitment, clonal expansion, and early osteoblastic differentiation [26], [27], and is a direct upstream regulator of *NELL-1* gene expression [3]. Our previous studies have demonstrated that Runx2 directly activates *NELL-1* transcription by physically binding to OSE2 sites on its promoter region [3]. In this current study, reporter system assays confirmed that Osterix directly represses Runx2-induced *NELL-1* expression through binding of multiple Sp1 sites on its promoter. Mechanistically, by using CHIP-qPCR assay, we were able to demonstrate that there was no difference in Runx2 binding of *NELL-1* promoter OSE2 sites with and without Osterix forced expression. This demonstrates that Osterix-mediated down-regulation of *NELL-1* expression does not involve disruption of Runx2 binding of the *NELL-1* promoter OSE2 sites. Instead, we found that general transcription factor RNA polymerase II binding to the *NELL-1* promoter is significantly decreased when Osterix is overexpressed, which may interfere with initiation of *NELL-1* gene transcription [28]. However, the exact role Osterix plays, along with RNA polymerase II, in the negative regulation of *NELL-1* with and without Runx2 induction remains unclear and warrants further study. Notably, there has been no evidence to date that Osterix and Runx2 interact with each other directly to alter their DNA binding and promoter transactivating activities [26].

To determine how Osterix repressive transcriptional regulation of *NELL-1* affects its osteogenic activity, we performed *in vitro* osteoblastic differentiation studies with either overexpression or specific siRNA knockdown of Osterix in Saos2 as well as in normal primary human osteoblast cells. Expectedly, the mRNA expression of *NELL-1* was severely inhibited by overexpression of Osterix. Notably, *NELL-1* repression was associated with the early transient decrease of Ocn and Opn mRNA indicating some level of impairment of NELL-1 osteoinductive capacity. In line with these findings, over two fold upregulation of *NELL-1* mRNA along with increase of Opn at the early phase, and increase of Ocn and mineralization at the late stage of osteoblastic differentiation were observed after Osterix knock down by specific siRNA. Interestingly, the different pattern of *Opn* expression between Saos-2 osteosarcoma cells and normal primary human osteoblast cells suggests a more complicated role for *Osterix* in osteoblastic differentiation at different maturation stages of human osteoblasts. Taken together, these data definitively demonstrate the functional impact and significance of Osterix repression of *NELL-1*. Furthermore, the forced expression of *NELL-1* remarkably reduced Osterix mRNA levels in Saos-2 cells (data not shown), demonstrating reciprocal repression of Osterix by NELL-1. This further confirmed our previous study on MC3T3 cells that showed transduction of *AdNELL-1* inhibited Osterix mRNA expression without affecting Runx2 mRNA levels [5], [16].

The repressive regulation of NELL-1 by Osterix may seem paradoxical given that both are known to be pro-osteoblastic, with many reports having shown that Osterix and NELL-1 can positively regulate osteoblast differentiation [5]–[7], [9]–[11]. However, in reality, this is not uncommon. For instance, Osterix, a pro-osteogenic regulator, negatively regulates the Wnt signaling pathway which is known to play a crucial role in the control of bone mass [22]. Osterix inhibits the Wnt signaling pathway through several mechanisms, including binding to and activating the Wnt antagonist DKK1 promoter, or interrupting TCF binding to its DNA elements and then suppressing downstream β-catenin activity [22].

Studies on the inter-relationship among various factors involved in the transcriptional regulatory network of osteogenesis are few in number and provide only limited answers likely owing to the high complexity of this area of study. What is known is that NELL-1 is a critical component in regulating osteoblastic differentiation, and that both Runx2 and Osterix are involved in its transcriptional regulation and osteogenic function. Runx2, a positive regulator of NELL-1, is highly expressed during transition from mesenchymal cells to preosteoblasts and immature osteoblasts [26]. NELL-1 may be an effector of a large portion of Runx2′s role, as it is a key downstream functional mediator in this process [7]. Osterix negative regulation of NELL-1, which is also tightly regulated by Runx2, may result from a delicate balancing of various driving forces in this regulatory network, modulating *NELL-1* expression levels as needed at different developmental time points. Moreover, the overexpression of *NELL-1* also affects Runx2 expression levels or bioactivity reciprocally, adding to the complexity of the regulatory network (**Fig. 6C**).

We expect that the regulatory relationship between NELL-1 and Osterix presented here from our *in vitro* studies is likely also true *in vivo*. Our preliminary studies revealed a higher level of Osterix expression with less mineralization in neonatal calvarial bones in ENU-induced *Nell-1* deficient mice compared to that of wild type mice suggesting existence of a similar regulatory relationship between Nell-1 and Osterix *in vivo* (data not shown). More extensive investigation of mouse skeletal development is needed for conclusive results, and is an area of future study for us.

Collectively, our findings through investigation of Osterix transcriptional regulation of and functional impact on *NELL-1* represent further understanding of the complex regulatory network that governs osteochondrogenesis.
